# Frontiers in Brain Imaging Methods Grand Challenge

**DOI:** 10.3389/fnins.2012.00096

**Published:** 2012-07-06

**Authors:** Jean-Baptiste Poline, Russell A. Poldrack

**Affiliations:** ^1^Neurospin, Institut d’Imagerie Biomédicale, Commissariat à l’Energie Atomique et aux Energies AlternativesGif sur Yvette, France; ^2^Henry H. Wheeler, Jr. Brain Imaging Center, University of California at BerkeleyBerkeley, CA, USA; ^3^Imaging Research Center, University of Texas at AustinAustin, TX, USA

The use of brain imaging techniques in research has grown rapidly during the past 20 years across a broad range of fields, including medicine, neuroscience, psychology, economics, and philosophy. This growth is unlikely to slow down. At the heart of this growth lies the development of brain imaging methods, including new methods for data acquisition, new approaches for the modeling and analysis of data, and new software tools. Methods in this field, as in others, are core to our scientific activity.

As these methods are becoming more sophisticated and require more expert knowledge, advances and discoveries are also becoming increasingly interdisciplinary, with contributions from a range of domains including physics, statistics, neuroscience, computer science, genetics, and psychology. This combination of increased technical complexity and growing need for interdisciplinary research poses new challenges to our community.

Concurrently, there is a growing concern about the reliability of published research findings in neuroimaging. This concern started with high-profile papers criticizing the use of circular analysis techniques in fMRI analysis (Kriegeskorte et al., 2009; Vul et al., 2009), and the discussion has become particularly heated in the blogosphere^1^. Concerns about the reliability of neuroimaging results arise largely by the great degree of analytic flexibility that is afforded by both existing and emerging analysis tools, and have led to an increased awareness and focus on the need to ensure reproducibility of research results.

In the following, we outline what we see as the major challenges to be met by the field of neuroimaging. A new era must arise in brain imaging, putting greater emphasis on the factors that will improve the reproducibility and reliability of research results, and thus on methods that ensure optimal outcomes. We identify below several directions that we hope to push forward in the publication of brain imaging methods. In line with the principles of the Frontiers journals, we propose that openness (in code, data, and access) is of fundamental importance.

## Methods that Matter

Methods for data acquisition and analysis are and will increasingly be central to scientific research using brain imaging. However, some methods matter more than others, either because they have a strong impact on the way we acquire, process, manage, interpret or reuse the data, or because they are more principled or mathematically grounded. Too often the impact of a new method is poorly assessed, and new methods are often developed that are unlikely to be applicable to actual studies. We hope to see a greater focus on the impact of improvements of acquisition and processing methods, and more work on how to make these methods accessible to the scientific community.

## Open Data and Code

Openly sharing all of the materials that go into a study, including data and code, is not only the way to make fair and informed judgment on the quality of a work, but it would dramatically increase the pace of progress and the reliability of results. Even more importantly, it should be thought of as a fundamental principle on how to conduct research: if we believe that research is about advancing knowledge in our society, then making data and code available is not only a way to allow a strong review mechanism but a principled necessity for scientific progress. In this respect, the development of the software tools which permit such sharing and collaboration or the description of new ways for using these tools will receive high priority in this new journal. We also believe that the details of methods matter a great deal, and we will strive to find ways to accommodate the most detailed methods descriptions possible.

## More Models

Models are the formalized representations of knowledge (often using the language of mathematics) with which hypotheses can be more formally specified and detailed predictions can be made. We hope to see more publications on the description and implementation of models, comparison between modeling approaches, and the use of improved models to obtain more accurate predictions. These models should ideally be developed and tested with large sets of openly accessible data.

## Educating Scientists

The continued progress of neuroimaging research relies upon the adequate training of each new generation of students in the latest techniques as the field evolves. We encourage submission of papers that describe new approaches for educating researchers in the latest methods or that propose tools or materials that will be useful in this regard.

## Conclusion

We believe that a publication, rather than being viewed as a static document, should be seen as a basis for dialog as well as for further research. The Frontiers publication mechanism provides the basis for such dialog, and we hope to further enhance this by the development of mechanisms for the open sharing of code and data. We hope that Frontiers in Brain Imaging Methods will become the premier outlet for publications related to brain imaging methods and foster a more fair and open research spirit.

## Footnotes

^1^http://www.danielbor.com/dilemma-weak-neuroimaging/
